# Longitudinal assessment of quality of life and symptom burden in locally advanced rectal cancer patients receiving IMRT-based preoperative radiotherapy: A prospective cohort study

**DOI:** 10.1007/s00384-025-05019-0

**Published:** 2025-12-02

**Authors:** Christina G. Truelsen, Camilla S. Kronborg, Anne Ramlov, Christian A. Hvid, Karen-Lise G. Spindler

**Affiliations:** 1 Danish Centre for Particle Therapy, Aarhus University Hospital, Aarhus, Denmark; 2 Department of Oncology, Aarhus University Hospital, Aarhus, Denmark; 3 Department of Clinical Medicine, Aarhus University, Aarhus, Denmark

**Keywords:** Rectal cancer, Neoadjuvant radiation, Patient-reported outcomes, EORTC QLQ-C30

## Abstract

**Purpose:**

Preoperative radiotherapy (pRT) for rectal cancer (RC) reduces local recurrence rates. However, treatment-induced side effects may compromise patient-reported quality of life (QoL). This study aimed to report longitudinal QoL and physician-assessed toxicity in RC patients receiving preoperative intensity modulated radiotherapy (IMRT).

**Methods:**

This prospective cohort study included 123 RC patients treated with short-course (SCRT) or long-course chemoradiotherapy (LCRT). Patient-reported outcomes (PRO) were assessed using the EORTC QLQ-C30 and CR29 questionnaires at pretreatment, end of treatment, preoperatively, and at 1-year follow-up. Physician-reported toxicity was evaluated using Common Terminology Criteria for Adverse Events (CTCAE). Longitudinal changes in PROs were analysed using mixed-effects regression modelling. CTCAE grades were reported as frequencies, and symptom transitions illustrated using Sankey diagrams.

**Results:**

For EORTC C30 items, pRT-induced transient declines were observed for Global Health, physical, role and social functioning, fatigue, and pain, with scores recovering at preoperative assessment, except for persistent worsening for fatigue. At 1-year, Global Health remained stable; emotional functioning improved; fatigue and social functioning showed minor persistent worsening. Bowel and bladder symptoms peaked during pRT and gradually resolved or improved at 1Y. CTCAE grades were predominantly mild; diarrhoea and rectal bleeding improved over time, while urinary dysfunction and fatigue increased modestly. Sankey plots illustrate symptom transitions. Discrepancies were noted between physician- and patient-reported outcomes.

**Conclusion:**

IMRT-based pRT was associated with largely preserved QoL at 1Y. Reported trajectories of PRO and CTCAE scores provide complementary insights to support physician–patient communication, with differences underlining the importance of integrating both perspectives.

**Supplementary Information:**

The online version contains supplementary material available at 10.1007/s00384-025-05019-0.

## Introduction

For locally advanced rectal cancer (LARC), multimodal treatment involving preoperative radiotherapy (pRT) has significantly reduced the risk of local recurrence. However, multimodality treatment approaches come with the risk of treatment-related side effects.

In recent decades, the multidimensional concept of quality of life (QoL) has gained prominence, with the European Organisation for Research and Treatment of Cancer (EORTC) developing a modular approach to assess QoL in cancer patients. This includes the validated QLQ-C30 questionnaire for overall QoL and the disease-specific CR29 module for gastrointestinal symptoms [1–4]. These standardised instruments allow for consistent reporting and comparisons of patient-reported functional and symptom scores, as well as comparisons with population-based norms.

Advances in modern radiotherapy techniques, such as intensity-modulated radiotherapy (IMRT) and volumetric modulated arc therapy (VMAT), enable highly conformal dose delivery to the target whilst simultaneously sparing doses to organs at risk (OAR). Compared to conventional radiotherapy, IMRT reduces both acute and late toxicity [5, 6], potentially leading to improved quality of life (QoL). Patient-reported QoL is essential when evaluating the balance between therapeutic efficacy and symptom burden. Prior studies have primarily assessed QoL after conventional radiotherapy [7–15]. Clinical practice guideline encourages the use of IMRT [16]. Recent randomised studies assessing QoL have used a mix of 3D-CRT and IMRT [17]. Limited randomised studies have evaluated QoL in rectal cancer with pure IMRT-based approaches [18, 19] Few prospective or survey-based studies have assessed QoL following purely IMRT-based radiotherapy, most included mixed techniques [20, 21]. Notably, Couwenberg et al. reported QoL using IMRT in all 156 patients [22]. In this study, IMRT-based pRT is exclusively utilised.

Our study contributes to existing evidence by evaluating QoL in a cohort undergoing IMRT-based pRT. Through longitudinal data prospectively collected using CTCAE and EORTC QLQ-C30 and CR29 questionnaires, we assess symptom trajectories and functional recovery in a standardised setting. By analysing score changes and transitions, we aim to provide insights that go beyond traditional frequency or group comparisons.

## Patients/material and methods

### Patients and treatment

Patient-reported QoL, functional outcomes, and physician-reported toxicity were assessed in a prospective cohort study. Eligible patients had histologically confirmed rectal adenocarcinoma, with indication for pRT per national Danish guidelines [23].

This included T4 tumours in the lower/middle rectum, T3 tumours in the lower rectum, or T3 tumours in the middle rectum with ≥ 5 mm invasion into the muscularis propria, tumour deposits, or nodes ≤ 2 mm from the mesorectal fascia (MRF) or circumferential resection margin (CRM). From 2024, revised criteria included tumours ≤ 10 cm from the anorectal junction or below the peritoneal reflection if MRI showed mrEMVI +, MRF ≤ 1 mm, tumour deposits, irregular nodes, or lateral pelvic nodes ≥ 8 mm, T4 tumours or T3 tumours with > 5 mm extramural invasion. Additionally, tumours above the peritoneal reflection if risk of non-radical resection or mrEMVI + [24]. Patients with oligometastatic disease considered for curative treatment were also included. Staging followed International Union for Cancer Controls TNM 8th edition. Participants were enrolled at Aarhus University Hospital between February 2017 and November 2024. Informed consent was provided by all participants.

Diagnostic work-up including endoscopy, pelvic MRI, CT scans of the thorax, abdomen, and pelvis, followed by multidisciplinary team evaluation. The gross tumour volume (GTV-T) was contoured on CT with MRI guidance. The clinical target volume (CTV-T) included GTV-T and the rectal circumference at tumour level, with a 5 mm isotropic margin (10 mm cranio-caudally), adjusted for anatomical barriers. The internal target volume (ITV-T) was generated by expanding CTV-T (7 mm cranio-caudally/laterally, 4 mm posteriorly, 10 mm anteriorly) while adjusting for rigid structures. CTV-E encompassed mesorectal, presacral, and lateral lymph nodes, including CTV-T. Selective nodal volumes (external iliac, inguinal, ischioanal) were added based on tumour invasion. ITV-E had no cranio-caudal/lateral margins, with optional ventral expansion (5–20 mm) toward the bladder. For both PTV-T and PTV-E, margins for the 25 Gy/5F regimen were 5 mm in the lateral and anteroposterior directions and 8 mm cranio-caudally; for the 50.4 Gy/28F regimen, a uniform 5 mm isotropic margin was used. A modified volume could be chosen for a conservative SCRT approach with GTV-T including the primary tumour; CTV encompassing tumour and mesorectum (20 mm cranio-caudally) and, where appropriate, adjacent organ invasion with a 1.5 cm margin. ITV = CTV + 5 mm (adjusted anatomically); PTV = ITV + 5 mm. OARs were delineated per RTOG guidelines [25].

pRT was delivered using IMRT/VMAT (a standard of VMAT 2 arcs) with either a short-course (SCRT) regimen of 25 Gy in 5 fractions or a long-course (LCRT) regimen of 50.4 Gy in 28 fractions, the latter combined with capecitabine 850 mg/m^2^ BID. Surgery, adhering to national guidelines, was performed 6–8 weeks after radiotherapy.

The study adhered to the principles of the Helsinki Declaration and was approved by the Danish Data Protection Agency and the Regional Committee on Health Research Ethics (M-2016–63-16).

### QoL- and toxicity assessment

The primary endpoint was the longitudinal assessment of PRO measures (PROMS) to monitor QoL in patients undergoing pRT for rectal cancer according to national Danish guidelines. PROMs were assessed using the validated cancer-specific core questionnaire EORTC QLQ-C30 [1, 2] and colorectal cancer module QLQ-CR29 [3, 4], at pretreatment (PT), end of pRT (EOT), 4–6 weeks post-pRT (pre-operatively; PO), and 1-year follow-up (1Y). Questionnaires were administered in-clinic (paper format) and mailed at PO and 1Y follow-up. Concurrently, physician-reported toxicities were evaluated using the 4th edition of National Cancer Institute Common Terminology Criteria for Adverse Events (NCI-CTCAE), which was collected as a predefined set of variables during in-clinic visits and via phone calls at PO and 1Y.

### Statistics

Clinicopathological characteristics between LCRT and SCRT data completion rates were compared using an unpaired t-test, Chi-square test, or Fisher's exact test, when appropriate (P < 0.05). PROMs from the EORTC QLQ-C30 and CR29 questionnaires were processed according to the EORTC guidelines [26], generating linear transformed scores (0–100), where higher functional scores indicate better function and higher symptom scores reflect greater symptom burden. QLQ-C30 domains were compared to Danish general population reference values matched for age- and sex, provided in the article by Juul et al. [27], with reference values weighted to reflect study demographics, reported as actual mean ± 1 standard deviation (SD). Longitudinal changes were analysed using linear mixed-effects regression models (LMMs), with random intercepts for patients to account for within-subject clustering. An unstructured residual covariance matrix was specified across time points, allowing each pairwise residual correlation and variance to be estimated, without assuming any specific correlation structure over time, while assuming missing data were missing at random. The models were specified without a fixed intercept to estimate separate means for each time point directly. Predicted means with 95% confidence intervals (CIs) were estimated using margins analysis. Mean differences over time were assessed using Wald tests based on the delta method, with results reported as 95% CIs and corresponding p-values. Analysis of the CR29 stoma care item failed to converge due to shifts in stoma status over time, preventing robust model estimation. Clinical interpretation of changes in score was reported per Osoba et al. [28]. Physician-reported toxicity was analysed as non-paired cohort frequencies, with toxicity grades assessed for a predefined set of NCI-CTCAE variables. Differences in NCI-CTCAE toxicity from PT to 1Y were assessed, applying the Wilcoxon signed-rank test with Benjamin-Hochberg correction. Multiple linear regression assessed the association between Global Health Score/QoL at 1Y and treatment modality, adjusting for age, sex, performance status, metastatic status, and 1Y stoma presence. A *p*-value ≤ 0.05 was considered statistically significant. Statistical analyses were conducted using Stata/IC18.0 (StataCorp LCC). The study was reported in accordance with the STROBE guidelines.

## Results

### Patient characteristics and assessment completion rates

**Fig. 1:**
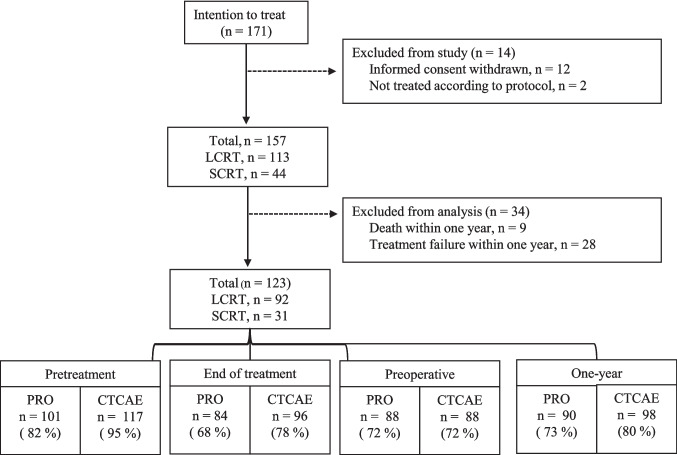
Patient flow and data completion rates for patient-reported outcomes EORTC QLQ-C30 and -CR29, and physician-reported NCI-CTCAE at given timepoints

Of 171 patients initially enrolled, 123 remained eligible for the primary QoL analysis after exclusion due to consent withdrawal, protocol deviations, or recurrence/death within one year. Among these, 92 (75%) received LCRT, and 31 (25%) received SCRT. The median age was 67 years (range: 31–94); 63% of patients were male, and 70% had performance status 0. Most had T3 tumours, 73% were node positive, and 7% had oligometastatic M1 disease, treated with a curative intent. Patient and treatment characteristics are presented in Table 1. Patients treated with SCRT had significantly older age, a poorer performance status, and a greater prevalence of nodal involvement and M1 disease, aligning with treatment guidelines. Prior to treatment, 101 patients (82%) completed PRO questionnaires, and 117 patients (95%) had complete NCI-CTCAE assessment. A patient flow diagram with data completion rates is presented in Fig. 1. No major clinicopathological differences were observed between PRO responders and non-responders at pretreatment or at 1Y.

**Table 1:** Clinicopathological patient characteristics

Clinicopathological characteristics	Total cohort, N (%)	LCRT, N (%)	SCRT, N (%)	*P*-value
No. (%)	123 (100%)	92 (75%)	31 (25%)	
Age, median(range),y	67 (31–94)	65 (31–82)	75 (43–94)	** < 0.01**
Sex				
Female	45 (37%)	37 (40%)	8 (26%)	0.15
Male	78 (63%)	55 (60%)	23 (74%)	
Performance status*				** < 0.01**
0	86 (70%)	71 (77%)	15 (48%)	
1	36 (29%)	21 (23%)	15 (48%)	
2	1 (1%)	0 (0%)	1 (3%)	
BMI, median(range)	26 (17.3–42.2)	26.1(17.3–40.1)	25.2 (17.7–42.2)	0.84
Distance from anorectal verge:				0.66
0–5 cm	56 (46%)	41 (45%)	15 (48%)	
5–10 cm	57 (46%)	42 (46 %)	15 (48%)	
10–15 cm	10 (8%)	9 (10%)	1 (3%)	
cT stage				0.94
T2	10 (8%)	7 (8%)	3 (10%)	
T3	90 (73%)	67 (73%)	23 (74%)	
T4	20 (16%)	15 (16%)	5 (26%)	
NA**	3 (2%)	3 (3%)	0 (0%)	
cN stage				** < 0.01**
N0	35 (28%)	23 (25%)	12 (39%)	
N1	39 (32%)	25 (27%)	14 (45%)	
N2	48 (39%)	43 (47%)	5 (16%)	
NA**	1 (1%)	1 (1%)	0 (0%)	
cM stage				** < 0.01**
M0	115 (94%)	90 (98%)	25 (81%)	
M1	8 (6%)	2 (2%)	6 (19%)	
Operative technique				0.06
Low anterior resection	32 (26 %)	27 (29%)	5 (16%)	
Abdominoperineal resection	84 (68 %)	60 (65%)	24 (77%)	
Other	4 (3%)	4 (4%)	0 (0%)	
Not operated†	3 (2%)	1 (1%)	2 (6%)	

^*^Eastern Cooperative Oncology Group performance status score. **Patients included with local recurrence after transanal endoscopic operation, where TNM-stage is not routinely applicable. † Either due to patient wish or WW: watch-and-wait approach

### Patient-reported quality of life

**Fig. 2:**
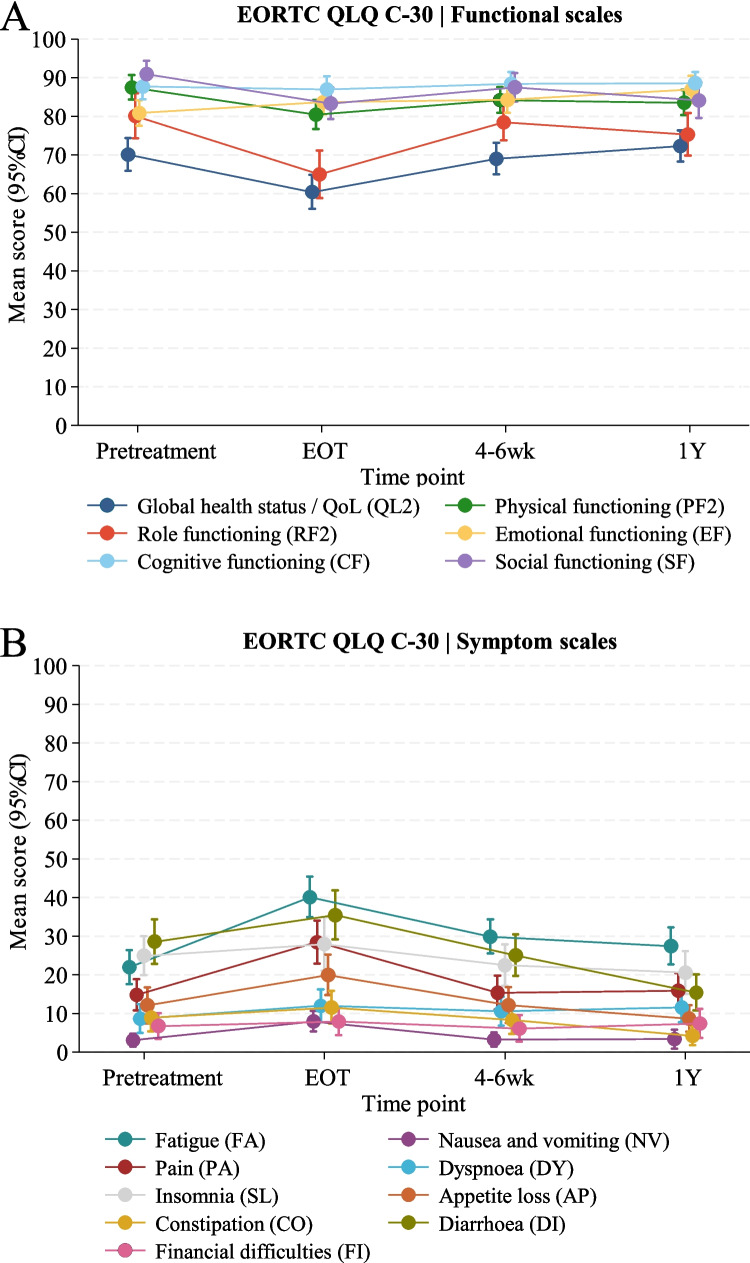
EORTC QLQ C-30 functional (**A**) and symptom (**B**) scales. Scores are presented at given timepoints as mean score with 95% confidence intervals derived by linear mixed model analysis. Higher functional scores indicate better functioning, whereas higher symptom scores reflect greater symptom burden severity

The EORTC QLQ-C30 functional- and symptom domains, derived from linear mixed model analysis, are depicted in Fig. 2, with score differences and corresponding clinical relevance presented in Table 2. The analysis of EORTC QLQ-C30 scores demonstrated statistically significant declines from PT to EOT, with minor declines in Global health status, physical and social functioning, appetite loss, and diarrhoea, and more pronounced deterioration observed for role functioning, fatigue, and pain. Four to six weeks post-radiation, most scores had stabilised relative to PT, except for fatigue, which remained slightly impaired (7.9 points; 95% CI: 3.5 to 12.3, *p* < 0.001). At 1-year follow-up, Global Health Score remained stable compared to PT, indicating preserved overall QoL. Among the other functional scales, minor improvement was noted for emotional functioning (6.1 points; 95% CI: 1.8 to 10.5, *p* < 0.01), whereas minor worsening was reported for social functioning (−6.8 points; 95% CI: −11.6 to −2.0, *p* < 0.01). Regarding symptom scales from PT to 1Y, minor worsening was observed for fatigue (5.5 points; 95% CI: 0.8 to 10.1, *p* = 0.02), whereas moderate improvement was noted for diarrhoea (−13.2 points; 95% CI: −19.7 to −6.6, *p* < 0.0001). All other domains remained unaffected. Although physical functioning declined (−3.9 points; 95% CI: −7.0 to −0.9, *p* = 0.01), it was classified as *no change* due to the small score difference, indicating minimal clinical relevance. Thus, while transient declines were observed during treatment, most domains recovered, with stable overall health at 1Y. However, slight residual effects on fatigue, social and physical functioning persisted. The predicted mean scores for EORTC QLQ-C30 and -CR29 are reported for each time point in Supplementary Tables 1 and Table 2, respectively. In multivariate analysis, age was positively associated with higher Global Health Score/QoL score at 1Y (β = 0.84, 95% CI: 0.49 to 1.19, *p* < 0.001), while poorer performance status was significantly associated with lower Global Health Score/QoL score at 1Y (β = −21.08, 95% CI: −29.69 to −12.46, *p* < 0.001). Treatment modality (LCRT vs. SCRT), sex, and stoma presence were not significant predictors of Global Health Score/QoL, see Supplementary Table 3. Subgroup analysis revealed that diarrhoea scores were significantly higher in non-stoma patients (*p* = 0.003), while stoma patients had increased fatigue at 1Y (*p* = 0.03). The EORTC QLQ-C30 functional scores did not differ significantly from those of an age and sex matched Danish reference cohort, illustrated in Supplementary Fig. 1.

**Table 2:** EORCT-QLQ C30 score differences estimated by linear mixed model analysis

EORTC QLQ-C30		Pretreatment vs. end of treatment			Pretreatment vs. preoperative			Pretreatment vs. one-year		
		Difference: mean (95% CI)	Group change†)	*P*-value	Difference: mean (95% CI)	Group change†	*P*-value	Difference: mean (95% CI)	Group change†	*P*-value
Q^a^	**Functional scales**^**b**^									
29, 30	Global health status	−9.7 (−13.8;−5.6)	Minor worsening	< 0.0001	−1.1 (−5.1;2.9)	No change	0.60	2.2 (−2.5;6.9)	No change	0.36
1–5	Physical functioning	−7.0 (−10.2;−3.8)	Minor worsening	< 0.0001	−3.3 (−5.9;−0.7)	No change	0.01	−3.9 (−7.0;−0.9)	No change	0.01
6, 7	Role functioning	−15.1 (−20.1;−10.2)	Moderate worsening	< 0.0001	−1.6 (−6.6;3.4)	No change	0.53	−4.8 (−11.1;1.4)	No change	0.13
21–24	Emotional functioning	2.9 (−0.1;5.9)	No change	0.06	3.4 (0.1;6.7)	No change	0.04	6.1 (1.8;10.5)	Minor improvement	< 0.01
20, 25	Cognitive functioning	−0.8 (−4.3;2.8)	No change	0.67	0.7 (−2.3;3.6)	No change	0.65	0.9 (−2.3;4.1)	No change	0.60
26, 27	Social functioning	−7.6 (−11.1;−4.2)	Minor worsening	< 0.0001	−3.4 (−7.2;0.3)	No change	0.07	−6.8 (−11.6;−2.0)	Minor worsening	< 0.01
	**Symptom scales**^**c**^									
10, 12, 18	Fatigue	18.1 (13.7;22.5)	Moderate worsening	< 0.0001	7.9 (3.5;12.3)	Minor worsening	< 0.001	5.5 (0.8;10.1)	Minor worsening	0.02
14, 15	Nausea and Vomiting	4.9 (2.2;7.6)	No change	< 0.001	0.1 (−1.2;1.5)	No change	0.83	0.3 (−2.4;3.1)	No change	0.82
9, 19	Pain	13.6 (8.8;18.5)	Moderate worsening	< 0.0001	0.6 (−3.7;4.9)	No change	0.79	1.1 (−3.8;6.0)	No change	0.66
8	Dyspnoea	3.3 (−1.0;7.7)	No change	0.13	1.9 (−1.7;5.6)	No change	0.30	2.9 (−1.1;6.9)	No change	0.15
11	Insomnia	3.1 (−2.5;8.6)	No change	0.28	−2.4 (−7.4;2.6)	No change	0.34	−4.3 (−10.3;1.6)	No change	0.15
13	Appetite loss	7.9 (3.0;12.8)	Minor worsening	< 0.01	0.1 (−4.8;5.0)	No change	0.98	−3.4 (−7.9;1.0)	No change	0.13
16	Constipation	2.6 (−1.5;6.8)	No change	0.22	−0.6 (−4.0;2.8)	No change	0.73	−4.7 (−7.7;−1.7)	No change	< 0.01
17	Diarrhoea	6.9 (0.2;13.6)	Minor worsening	0.04	−3.5 (−9.6;2.5)	No change	0.25	−13.2 (−19.7;−6.6)	Moderate improvement	< 0.0001
28	Financial difficulties	1.2 (−1.5;3.9)	No change	0.39	−0.6 (−2.9;1.6)	No change	0.58	0.7 (−3.6;4.9)	No change	0.76

^a^Q: Question numbers refer to the EORTC QLQ-C30 module. ^b^Functional scales: Higher scores denote improved functioning. ^c^Symptom scales: Elevated scores indicate increased symptom severity. † Group changes represent score changes from 0–4 points = no change. 5–10 points = minor. 11–20 points = moderate. > 20 = major

**Fig. 3:**
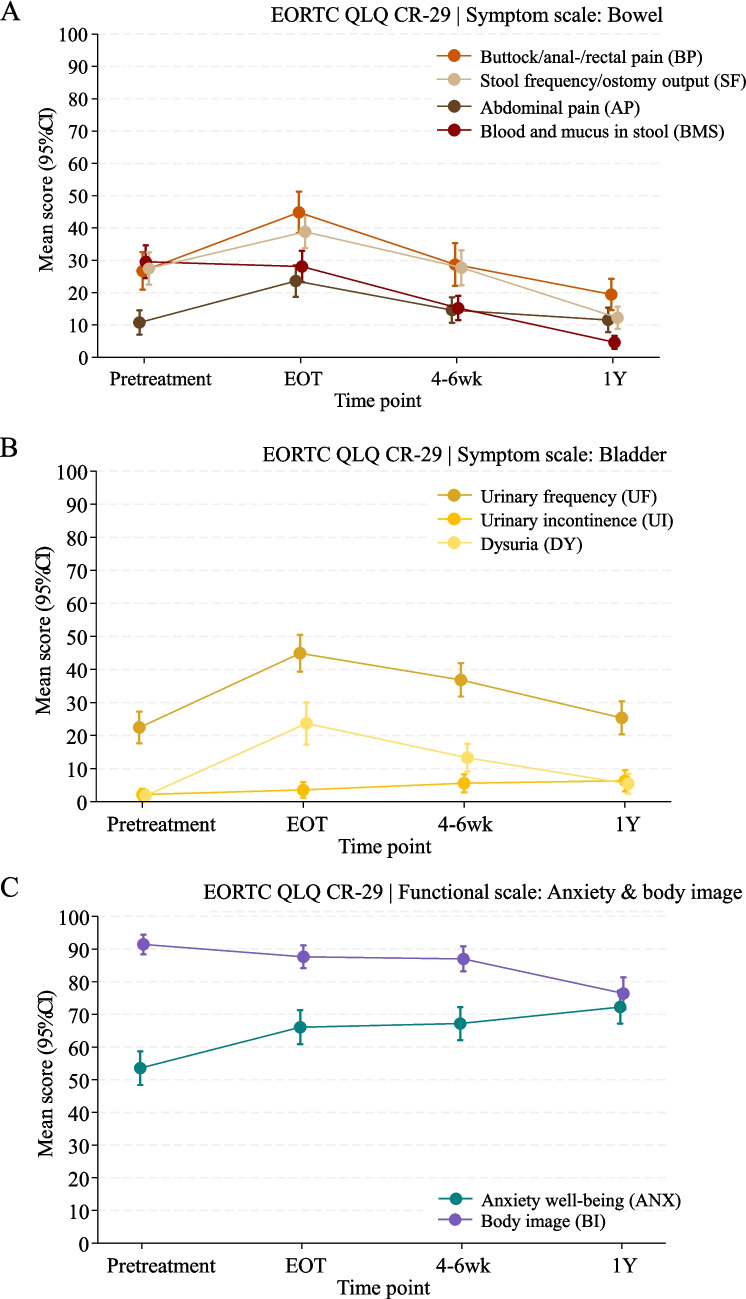
Selected EORTC QLQ CR-29 items, grouped by bowel (**A**), bladder (**B**), and psychosocial (**C**) domains, estimated via a linear mixed model analysis. Bowel and bladder items are symptom-based items, with higher scores indicating greater symptom burden. Conversely, anxiety and body image are functional items, where higher scores denote better psychosocial functioning

The EORTC QLQ-CR29 module, assessing bowel and bladder symptoms, with score changes estimated by linear mixed models, is presented in Table 3. For the bowel-related items, significant symptomatic trajectories were noted over time, illustrated in Fig. 3A. The EORTC QLQ-CR29 module adjusts items based on stoma status. As stoma prevalence increased from 4% at PT to 85% at 1Y, combined scores are reported for stoma-related items, ensuring longitudinal comparability despite shifts in stoma status. Abdominal pain, buttock/rectal/anal pain, bloating, sore skin and stool frequency/ostomy output significantly intensified during treatment but gradually improved, with buttock/rectal/anal pain and stool frequency/ostomy output showing minor to moderate improvement at 1Y compared to PT (−7.3 points; 95% CI: −14.1 to −0.5, *p* = 0.04 and −15.2 points; 95% CI: −20.4 to −10.0, *p* < 0.0001, respectively). Treatment did not significantly exacerbate faecal incontinence or flatulence. Conversely, blood and mucus in stool continuously improved, reaching major improvement at 1Y (−24.9 points; 95% CI: −30.4 to −19.4, *p* < 0.0001).

**Table 3:** EORCT-QLQ CR29 score differences estimated by linear mixed model analysis

EORTC QLQ-CR29		Pretreatment vs. end of treatment			Pretreatment vs. preoperative			Pretreatment vs. one-year		
		Difference mean (95% CI)	Group change†)	*P*-value	Difference mean (95% CI)	Group change†	*P*-value	Difference mean (95% CI)	Group change†	*P*-value
Q^a^	**Functional scales**^**b**^									
43	Anxiety	12.5 (7.5;17.5)	Moderate improvement	< 0.0001	13.7 (8.3;19.0)	Moderate improvement	< 0.0001	18.7 (12.6;24.8)	Moderate improvement	< 0.0001
44	Weight	−2.4 (−6.7;1.9)	No change	0.27	−3.2 (−7.5;1.0)	No change	0.14	−5.3 (−11.2;0.7)	Minor worsening	0.08
45–47	Body image	−3.8 (−7.3;−0.3)	No change	0.03	−4.4 (−7.9;−1.0)	No change	0.01	−15.0 (−19.8;−10.2)	Moderate worsening	< 0.0001
56	Sexual interest (men)	−4.6 (−11.6;2.4)	No change	0.20	−1.4 (−7.6;4.7)	No change	0.65	3.5 (−2.6;9.6)	No change	0.26
58	Sexual interest (women)	−6.0 (−11.0;−1.0)	Minor worsening	0.02	−1.9 (−8.7;4.9)	No change	0.58	0.0 (−7.5;7.5)	No change	0.99
	**Symptom scales**^**c**^									
31,32	Urinary frequency	22.5 (17.1;27.8)	Major worsening	< 0.0001	14.4 (8.6;20.2)	Moderate worsening	< 0.0001	2.9 (−3.2;9.1)	No change	0.35
33	Urinary incontinence	1.4 (−1.1;3.9)	No change	0.26	3.4 (0.6;6.3)	No change	0.02	4.2 (0.4;8.0)	No change	0.03
34	Dysuria	21.8 (15.7;28.0)	Major worsening	< 0.0001	11.5 (7.1;15.8)	Moderate worsening	< 0.0001	3.6 (0.1;7.0)	No change	0.04
35	Abdominal pain	12.8 (7.9;17.8)	Moderate worsening	< 0.0001	3.8 (−0.1;7.8)	No change	0.05	0.8 (−3.5;5.2)	No change	0.72
36	Buttock pain	18.1 (11.7;24.6)	Moderate worsening	< 0.0001	2.0 (−4.6;8.5)	No change	0.55	−7.3 (−14.1;−0.5)	Minor improvement	0.04
37	Bloating	10.4 (5.8;14.9)	Minor worsening	< 0.0001	2.7 (−2.4;7.8)	No change	0.30	−2.5 (−6.4;1.4)	No change	0.20
38,39	Blood and mucus in stool	−1.5 (−7.8;4.8)	No change	0.65	−14.3 (−19.7;−8.9)	Moderate improvement	< 0.0001	−24.9 (−30.4;−19.4)	Major improvement	< 0.0001
40	Dry mouth	12.2 (7.9;16.6)	Moderate worsening	< 0.0001	8.9 (4.8;12.9)	Minor worsening	< 0.0001	4.0 (−0.5;8.5)	No change	0.08
41	Hair loss	0.7 (−0.7;2.2)	No change	0.32	2.6 (0.5;4.6)	No change	0.01	1.9 (0.3;3.5)	No change	0.02
42	Taste problems	8.8 (4.9;12.7)	Minor worsening	< 0.0001	8.2 (4.7;11.8)	Minor worsening	< 0.0001	5.5 (1.6;9.4)	Minor worsening	0.01
49,49 s	Flatulence*	1.9 (−3.6;7.4)	No change	0.49	−1.3 (−7.8;5.2)	No change	0.70	−8.6 (−15.6;−1.7)	Minor improvement	0.02
50, 50 s	Faecal incontinence*	3.5 (−2.1;9.1)	No change	0.22	0.7 (−5.6;6.9)	No change	0.83	−0.7 (−6.3;4.9)	No change	0.81
51, 51 s	Sore skin*	21.6 (14.5;28.6)	Major worsening	< 0.0001	5.3 (−1.6;12.3)	Minor worsening	0.13	0.8 (−4.9;6.5)	No change	0.78
52, 53, 52 s, 53 s	Stool frequency/ ostomy output*	11.4 (6.5;16.2)	Moderate worsening	< 0.0001	0.2 (−5.6;6.1)	No change	0.93	−15.2 (−20.4;−10.0)	Moderate improvement	< 0.0001
54, 54 s	Embarrassment of bowel movement/ stoma*	1.8 (−3.0;6.7)	No change	0.46	0.7 (−4.4;5.7)	No change	0.80	7.3 (0.8;13.7)	Minor worsening	0.03
55	Stoma care problems	-	-	-	-	-	-	-	-	-
57	Impotence (men)	8.0 (−3.3;19.2)	Minor worsening	0.17	9.8 (2.5;17.2)	Minor worsening	< 0.01	35.3 (23.7;47.0)	Major worsening	< 0.0001
59	Dyspareunia (women)	6.9 (−3.6;17.5)	Minor worsening	0.20	15.6 (4.4;26.9)	Moderate worsening	< 0.01	17.9 (1.5;34.4)	Moderate worsening	0.03

^a^Q: question numbers refer to the EORTC QLQ-C30 module. ^b^Functional scales: Higher scores denote improved functioning. ^c^Symptom scales: Elevated scores indicate increased symptom severity. *These items in the EORTC QLQ-CR29 questionnaire are adapted questions designed to account for the presence or absence of a stoma. †Group changes represent paired score changes from 0–4 points = no change. 5–10 points = minor. 11–20 points = moderate. > 20 = major. -:The LMM analysis of stoma care problems failed to converge due to shifting stoma status causing unstable model estimation

Bladder-specific symptoms exhibited distinct patterns over time (Fig. 3B). Urinary frequency and dysuria worsened majorly from PT to the EOT. Both symptoms partially recovered by 4–6 weeks post-treatment and returned to PT levels at 1Y. No clinically relevant fluctuations in urinary incontinence were detected over time. These findings suggest that treatment-induced bladder dysfunction is primarily transient, peaking during treatment and gradually resolving thereafter.

Other significantly affected domains included anxiety, which demonstrated a moderate improvement across all time points, with a mean increase of 18.7 points (95% CI: 12.6 to 24.8, *p* < 0.0001) from PT to 1Y. In contrast, body image declined moderately over the same period (−14.4 points; 95% CI: −19.4 to −9.4, *p* < 0.0001), as displayed in Fig. 3C.

Sankey flow diagrams illustrating symptom trajectories and changes in reported severity for selected single-item raw EORTC scores are presented in Supplementary Fig. 2.

### Physician-reported toxicity and transition in symptoms

The predefined CTCAE variables were used to report non-paired frequencies. CTCAE grades 0–1 accounted for the largest proportion of observations, with only a minor proportion reaching grade 2–3 toxicity, presented in Supplementary Fig. 3.

From PT to 1Y, shifts in symptom frequencies were observed for several NCI-CTCAE categories. Improvements were seen in constipation, diarrhoea, anorexia, bloating, and rectal/stool haemorrhage. Among these, diarrhoea and rectal/stool haemorrhage showed statistically significant reductions. The most notable reduction was in rectal/stool haemorrhage, with any-grade rectal/stool haemorrhage decreasing from 57 to 6%, and (moderate-to-severe) grade 2–3 cases from 3 to 0%.

Conversely, increases were noted for fatigue, pain, nausea, vomiting, and urinary dysfunction. Fatigue became more prevalent, with any-grade cases increasing from 25% at PT to 40% at 1Y, while moderate-to-severe cases remained unchanged. Pain frequencies increased slightly (28% to 35%), with moderate-to-severe pain increasing from 5 to 7%. Regarding urinary symptoms, urinary frequency, incontinence, retention, and urgency all increased significantly, indicating greater urinary dysfunction at 1Y, see Supplementary Table 4. At 1Y, grade 3 toxicity was reported for constipation (1%) and diarrhoea (2%).

**Fig. 4:**
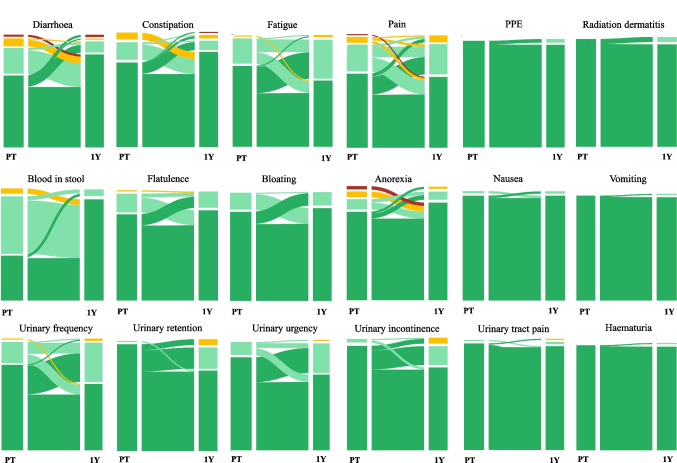
Sankey flow diagram for transition of physician-reported NCI-CTCAE symptom grades from pretreatment (PT) to 1 year after treatment (1Y). The left bar represents the distribution of NCI-CTCAE symptom scores at PT. The flows represent the transition of symptom grades from PT to 1Y. The right bar represents the distribution of symptoms at 1Y. The colours in the figure represent symptom grades as follows: grade 0 is depicted in dark green, grade 1 in light green, grade 2 in yellow, and grade 3 in red. *Abbreviations*: PPE: Palmar-plantar erythrodysesthesia syndrome

Sankey flow diagrams illustrate how symptoms change in severity from PT to 1Y for matched responses (*n* = 96). The Sankey flow diagrams, presented in Fig. 4, reveal patterns of resolution, persistence, and worsening that static proportions cannot capture. While proportions reflect symptom frequency, they do not convey the dynamic shifts in symptom burden, highlighting individual variations in symptom trajectories over time. For instance, at PT, 91% of patients reported CTCAE grade 0–1 diarrhoea, with 3% progressing to grade 2–3 by 1Y. Conversely, all patients with grade 2–3 diarrhoea at PT (9%) improved to grade 0–1 at 1Y.

## Discussion

This study provides a longitudinal assessment of QoL and toxicity in patients undergoing IMRT-based pRT for rectal cancer in a survivorship setting, offering insight into symptom burden and functional recovery within a standardised setting. Patients with treatment failure were excluded, as additional surgery, reirradiation, local metastasis-directed therapy, or palliative chemotherapy could influence scores. No significant clinicopathological differences were observed between responders and non-responders at PT or 1Y, although response bias remains a challenge in PRO studies.

EORTC QLQ-C30 analysis demonstrates that pRT impacts QoL, with the greatest declines reported for role functioning, fatigue, and pain. Most scores recovered by 4–6 weeks, except for minor persistent fatigue. At 1Y, Global Health remained stable, with a slight improvement in emotional functioning and minor worsening in social functioning, indicating overall preservation of QoL, despite acute declines during treatment.

While several studies have provided insights into QoL after rectal cancer treatment [7–10, 17, 20, 22], interstudy comparison is complicated by variations in surgical timing and approach, systemic therapy integration, and radiotherapy protocols. Randomised trials have compared QoL between SCRT and LCRT. In our study, no significant difference was found for Global Health Status/QoL between these modalities, corresponding with findings from the Polish Trial [8]. Similarly, the RAPIDO trial reported no compromise in QoL, bowel function, or increase in grade 3 toxicity, comparing SCRT + preoperative chemotherapy to LCRT [17]. The Dutch TME trial, comparing SCRT + TME to TME alone, showed similar long-term QoL but transient lower activity levels in the RT group at three months [7], aligning with the transient impairments in fatigue and physical functioning (although physical functioning scores did not reach clinical relevance, even though statistically significant) observed in our study at 4–6 weeks. Long-term follow-up in the Dutch trial revealed no differences in global health scores at 14 years, though SCRT + TME increased bowel dysfunction in non-stoma patients [29]. Among the cited studies, only the RAPIDO trial utilised both 3D-CRT and IMRT. Notably, this study solely relied on IMRT, setting it apart from earlier QoL studies that largely relied on conventional radiotherapy [7–14, 30, 31].

While radiotherapy and surgery increase bowel dysfunction [7–10, 32–34], longitudinal assessment is complicated by stoma status shifts. In this cohort, stoma prevalence rose from 4% at PT to 85% at 1Y, requiring grouping of QLQ-CR29 stoma-related items, potentially affecting bowel dysfunction interpretation.

Our findings indicate a transient increase in bowel dysfunction during pRT, followed by improvement at 1Y. While sphincter-preserving surgery avoids permanent stoma, it may cause faecal urgency and incontinence, characteristic of low anterior resection syndrome [35]. This raises the question of whether QoL is better in non-stoma versus stoma patients. In this study, stoma status did not significantly impact QoL, aligning with a review, reporting no consistent advantage for non-stoma patients [36]. In contrast, a population-based study observed worse QoL in stoma patients [34], while another study identified major LARS as the strongest negative QoL factor [37]. The Stockholm I and II trials found increased faecal and urinary incontinence in irradiated patients, with higher diarrhoea scores in non-stoma patients [11]. In this study, subgroup analysis revealed higher diarrhoea scores in non-stoma patients and greater fatigue in stoma patients at 1Y. This suggests that while stoma avoidance may preserve bowel function, it may come at the cost of other functional impairments.

Our findings reveal a discrepancy between patient- and physician-reported toxicity [38, 39]. While bladder-related PROs showed transient bladder dysfunction with recovery at 1Y, CTCAE-assessed toxicity persisted, possibly reflecting patients’ ability to adapt to persistent symptoms over time, contributing to improved perceived functioning despite ongoing clinical findings. This underscores the need for integrating both clinician and patient perspectives in functional outcome evaluations.

Sankey flow diagrams visualised symptom progression, capturing individual trajectories and shifts between groups, revealing both improvements in high-grade cases and worsening in mild cases [40]. While selection bias might be a limitation due to matched responses, presenting PRO in an intuitive format can enhance patient-physician communication on expected symptom development over time.

This study was conducted as a prospective descriptive analysis within a single-centre setting, which may limit external validity. The primary aim was to provide insight into patient-reported QoL rather than to formally test predefined effect sizes; results should therefore be interpreted with caution. Patients who experienced recurrence or death prior to follow-up were excluded, meaning the results reflect QoL among recurrence-free survivors and may be biased toward more favourable outcomes. Additional limitations include the 1-year follow-up period, which may not capture longer-term changes in QoL and late toxicities, the absence of a comparison group treated with alternative radiotherapy techniques, and the modest sample size, potentially limiting statistical power. No adjustment for multiple comparisons was performed, and p-values should therefore be interpreted with caution in light of the potential for inflated type I error.

Nevertheless, the prospective longitudinal design and standardised use of IMRT represent strengths of the study, addressing an important evidence gap in QoL research. They also support future research directions, including longer follow-up, multi-centre validation, or comparisons with a control group or proton therapy, and underscore the rationale for incorporating routine patient-reported outcome monitoring into follow-up protocols to enhance patient-centred care.

In conclusion, this study evaluates toxicity, QoL and functional recovery in LARC patients undergoing IMRT-based pRT according to national Danish guidelines. Despite transient declines, most domains recovered, ensuring reduced symptom burden and stable overall health at 1Y, providing information which may support clinicians in physician–patient communication and underlines the importance of both physician and patient-reported outcomes.

## Supplementary Information

Below is the link to the electronic supplementary material.

Supplementary file1 (PDF 18 KB)

Supplementary file2 (DOCX 1224 KB)

## Data Availability

The study data is stored in a Danish database. Data are not publicly available due to patient confidentiality and legal restrictions; the General Data Protection Regulation.
