# Supplementary material for: Longitudinal assessment of quality of life and symptom burden in locally advanced rectal cancer patients receiving IMRT-based preoperative radiotherapy: A prospective cohort study
Source: Int J Colorectal Dis. 2025 Dec 2;40(1):239. doi: 10.1007/s00384-025-05019-0 (PMC12672606; doi:10.1007/s00384-025-05019-0)
Supplement: Supplementary file 2 — Supplementary file2 (DOCX 1224 KB) [file 384_2025_5019_MOESM2_ESM.docx]

**Tables and Figures**

**Supplementary**

Supplementary Table 1: EORCT-QLQ C30 score at pretreatment, end of treatment, preoperative and one-year

Supplementary Table 2: EORCT-QLQ CR29 score at pretreatment, end of treatment, preoperative and one-year

Supplementary Table 3: Multiple linear regression

Supplementary Figure 1: EORTC QLQ-C30 Functional outcomes benchmarked against a Danish reference population

Supplementary Figure 2: Sankey Plots: patient-reported EORTC raw scores

Supplementary Figure 3: Physician-reported NCI-CTCAE toksixity reported for a predefined set of NCI-CTCAE variables

Supplementary Table 4: Differences in physician-reported NCI-CTCAE toxicity between pretreatment (PT) and one-year follow-up (1Y).

**Supplementary**

Table 1: EORCT-QLQ C30 score at pretreatment, end of treatment, preoperative and one-year by linear mixed model analysis

| **EORTC QLQ-C30** | |  |  |  |  |  |
| --- | --- | --- | --- | --- | --- | --- |
|  |  | ***Population** | **Pretreatment** | **End of treatment** | **Preoperative** | **One-year** |
|  |  | Actual mean (±1SD) | Predicted mean (95%CI) | Predicted mean (95%CI) | Predicted mean (95%CI) | Predicted mean (95%CI) |
| **Q^a^** | **Functional scales^b^** |  |  |  |  |  |
| 29, 30 | Global health status /QoL | 72.9 (49.8-96.0) | 70.1 (65.9-74.3) | 60.4 (56.1-64.8) | 69.0 (64.9-73.1) | 72.3 (68.3-76.3) |
| 1-5 | Physical functioning | 86.2 (67.9-100.0) | 87.5 (84.3-90.7) | 80.5 (76.8-84.2) | 84.2 (80.8-87.6) | 83.6 (80.4-86.8) |
| 6, 7 | Role functioning | 83.3 (57.2-100.0) | 80.1 (74.3-86.0) | 65.0 (58.8-71.2) | 78.5 (73.7-83.3) | 75.3 (69.8-80.8) |
| 21-24 | Emotional functioning | 85.2 (66.5-100.0) | 80.8 (77.5-84.2) | 83.7 (80.5-86.9) | 84.3 (80.9-87.7) | 87.0 (83.5-90.5) |
| 20, 25 | Cognitive functioning | 87.3 (69.0-100.0) | 87.7 (84.4-91.1) | 87.0 (83.6-90.3) | 88.4 (85.3-91.5) | 88.6 (85.7-91.5) |
| 26, 27 | Social functioning | 89.1 (67.7-100.0) | 91.0 (87.5-94.4) | 83.3 (79.2-87.4) | 87.5 (83.9-91.2) | 84.2 (79.5-88.8) |
|  | **Symptom scales^c^** |  |  |  |  |  |
| 10, 12, 18 | Fatigue | 22.8 (0.4-45.1) | 22.0 (17.6-26.4) | 40.1 (34.9-45.3) | 29.9 (25.5-34.3) | 27.4 (22.7-32.2) |
| 14, 15 | Nausea and Vomiting | 2.8 (0.0-12.7) | 3.0 (1.4-4.7) | 8.0 (5.4-10.6) | 3.2 (1.3-5.0) | 3.4 (0.9-5.8) |
| 9, 19 | Pain | 21.0 (0.0-47.0) | 14.8 (10.8-18.8) | 28.5 (22.9-34.0) | 15.4 (11.0-19.8) | 15.9 (11.4-20.4) |
| 8 | Dyspnoea | 12.2 (0.0-33.6) | 8.6 (4.9-12.4) | 12.0 (7.7-16.2) | 10.5 (6.8-14.3) | 11.6 (7.7-15.4) |
| 11 | Insomnia | 21.0 (0.0-48.0) | 24.9 (19.8-30.0) | 28.0 (21.1-34.9) | 22.5 (17.1-27.9) | 20.6 (15.1-26.1) |
| 13 | Appetite loss | 5.9 (0.0-22.8) | 12.1 (7.4-16.8) | 20.0 (14.7-25.2) | 12.2 (7.5-16.8) | 8.7 (5.3-12.0) |
| 16 | Constipation | 5.9 (0.0-21.6) | 8.9 (5.4-12.5) | 11.6 (7.3-15.8) | 8.3 (4.7-11.9) | 4.2 (1.7-6.7) |
| 17 | Diarrhoea | 6.6 (0.0-23.0) | 28.6 (22.9-34.3) | 35.5 (29.1-41.8) | 25.0 (19.7-30.4) | 15.4 (10.7-20.1) |
| 28 | Financial difficulties | 5.9 (0.0-23.4) | 6.7 (3.4-10.1) | 7.9 (4.4-11.5) | 6.1 (2.7-9.6) | 7.4 (3.7-11.1) |

^a^Q: question numbers refer to the EORTC QLQ-C30 module. ^b^Functional scales: Higher scores denote improved functioning. ^c^Symptom scales: Elevated scores indicate increased symptom severity. *A Danish reference population, matched for age and sex, derived from data by Therese Juul *et al*. (*Quality of Life Research*, 2014^1^). For this population, the *actual mean* is presented with standard deviation (±1 SD), while *predicted means* are estimated using mixed regression models and reported with 95% confidence intervals (CIs).

Table 2: EORCT-QLQ CR29 score at pretreatment, end of treatment, preoperative and one-year by linear mixed model analysis

| **EORTC QLQ-CR29** | | **Pretreatment** | **End of treatment** | **Preoperative** | **One-year** |
| --- | --- | --- | --- | --- | --- |
|  |  | Predicted mean (95%CI) | Predicted mean (95%CI) | Predicted mean (95%CI) | Predicted mean (95%CI) |
| **Q^a^** | **Functional scales^b^** |  |  |  |  |
| 43 | Anxiety | 53.5 (48.4-58.7) | 66.1 (60.9-71.3) | 67.2 (62.1-72.3) | 72.2 (67.2-77.3) |
| 44 | Weight | 89.1 (85.2-92.9) | 86.7 (82.2-91.1) | 85.9 (81.3-90.4) | 83.8 (79.1-88.5) |
| 45-47 | Body image | 91.4 (88.4-94.4) | 87.6 (84.1-91.1) | 87.0 (83.2-90.8) | 76.4 (71.5-81.3) |
| 56 | Sexual interest (men) | 31.9 (24.6-39.1) | 27.3 (20.3-34.2) | 30.5 (23.5-37.4) | 35.4 (28.9-41.9) |
| 58 | Sexual interest (women) | 16.1 (8.9-23.3) | 10.1 (4.0-16.3) | 14.2 (6.8-21.6) | 16.2 (9.0-23.3) |
|  | **Symptom scales^c^** |  |  |  |  |
| 31,32 | Urinary frequency | 22.4 (17.7-27.2) | 44.9 (39.4-50.5) | 36.8 (31.8-41.9) | 25.4 (20.4-30.4) |
| 33 | Urinary incontinence | 2.1 (0.3-3.9) | 3.6 (1.2-5.9) | 5.6 (2.8-8.3) | 6.3 (3.1-9.6) |
| 34 | Dysuria | 1.9 (0.2-3.5) | 23.7 (17.3-30.1) | 13.3 (9.1-17.5) | 5.4 (2.4-8.4) |
| 35 | Abdominal pain | 10.8 (7.0-14.5) | 23.6 (18.7-28.5) | 14.6 (10.7-18.6) | 11.6 (7.8-15.4) |
| 36 | Buttock pain | 26.7 (20.9-32.5) | 44.8 (38.4-51.3) | 28.7 (22.1-35.3) | 19.4 (14.6-24.3) |
| 37 | Bloating | 14.9 (10.5-19.2) | 25.2 (20.1-30.4) | 17.6 (12.5-22.6) | 12.4 (8.6-16.1) |
| 38,39 | Blood and mucus in stool | 29.5 (24.5-34.6) | 28.1 (23.2-32.9) | 15.2 (11.5-18.9) | 4.6 (2.6-6.7) |
| 40 | Dry mouth | 9.0 (5.8-12.3) | 21.3 (16.4-26.1) | 17.9 (13.3-22.5) | 13.0 (9.1-17.0) |
| 41 | Hair loss | 0.3 (-0.3-1.0) | 1.1 (-0.2-2.3) | 2.9 (1.0-4.8) | 2.2 (0.4-4.0) |
| 42 | Taste problems | 2.9 (0.9-4.9) | 11.7 (7.9-15.6) | 11.1 (7.5-14.7) | 8.4 (4.7-12.1) |
| 49,49s | Flatulence* | 26.5 (20.8-32.2) | 28.5 (23.4-33.6) | 25.2 (19.9-30.6) | 17.9 (13.7-22.1) |
| 50, 50s | Faecal incontinence* | 13.6 (9.6-17.7) | 17.2 (11.9-22.4) | 14.3 (9.6-19.0) | 12.9 (9.0-16.9) |
| 51, 51s | Sore skin* | 19.0 (14.0-24.1) | 40.6 (33.5-47.7) | 24.4 (18.5-30.3) | 19.9 (15.0-24.7) |
| 52, 53, 52s, 53s | Stool frequency/  ostomy output* | 27.5 (22.5-32.4) | 38.8 (33.7-43.9) | 27.7 (22.3-33.1) | 12.2 (8.8-15.7) |
| 54, 54s | Embarrassment of  bowel movement/stoma* | 10.7 (6.4-15.0) | 12.5 (8.1-17.0) | 11.4 (7.1-15.7) | 18.0 (12.2-23.7) |
| 55 | Stoma care problems | - | - | - | - |
| 57 | Impotence (men) | 31.8 (23.2-40.3) | 39.7 (29.2-50.2) | 41.6 (31.6-51.7) | 67.1 (56.4-77.7) |
| 59 | Dyspareunia (women) | 7.8 (-0.6-16.2) | 14.8 (4.3-25.3) | 23.5 (10.7-36.2) | 25.7 (12.7-38.8) |

^a^Q: question numbers refer to the EORTC QLQ-C30 module. ^b^Functional scales: Higher scores denote improved functioning. ^c^Symptom scales: Elevated scores indicate increased symptom severity. *These items in the EORTC QLQ-CR29 questionnaire are adapted questions designed to account for the presence or absence of a stoma. -:The LMM analysis of stoma care problems failed to converge due to shifting stoma status causing unstable model estimation.

Supplementary Table 3. Multiple linear regression assessing associations between Global Health Status/QoL at 1 year and patient/treatment characteristics

| **Variable** | **β (95 % CI)** | **p-value** |
| --- | --- | --- |
| Age at diagnosis | 0.84 (0.49 to 1.19) | <0.001 |
| Sex | 4.2 (–3.3 to 11.8) | 0.27 |
| Performance status | –21.1 (–29.7 to –12.5) | <0.001 |
| Treatment modality LCRT vs. SCRT | –2.8 (–12.6 to 7.1) | 0.58 |
| Stoma presence at 1Y | –3.5 (–13.4 to 6.4) | 0.49 |

Model fit: F(6, 83) = 6.68, p < 0.001, R² = 0.33

Figure 1: EORTC QLQ-C30 Functional outcomes, estimated as actual mean (±1 SD). The grey area depicts scores from an age- and sex-matched Danish reference population, presented as actual mean ±1 SD. Reference data is provided by Therese Juul *et al*. (*Quality of Life Research*, 2014).

Figure 2: Sankey flow diagram for transition of selected patient-reported outcomes from pretreatment (PT) to 1 year after treatment (1Y) for matched responses (n = 75, 60%). Single-item EORTC raw score trajectories, displayed in the Sankey plots, are reported separately to differentiate them from multi-item scores and linear mixed model estimates. The left bar represents the distribution of symptom scores at PT. The flows represent the transition of symptom scores from PT to 1Y. The right bar represents the distribution of symptoms at 1Y. The colours in the figure represent symptoms as follows: ´ *not at all* ´ is depicted in dark green, ´ *a little* ´ in light green, ´*quite a bit´* in yellow, and ´*very much´* in red.

Figure 3: Physician-reported NCI-CTCAE toxicity reported for a predefined set of NCI-CTCAE variables.


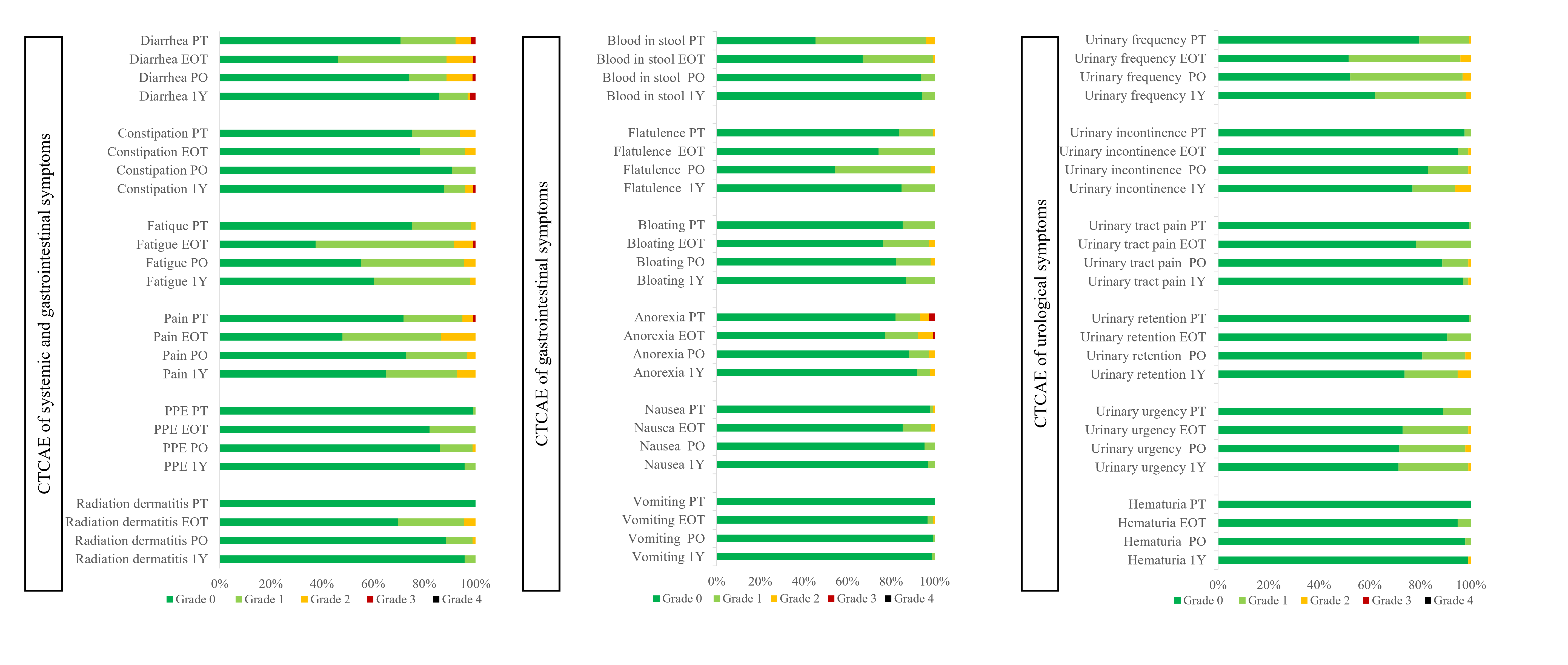


Abbreviations: PPE: Palmar-plantar erythrodysesthesia syndrome

Table 4: Differences in physician-reported NCI-CTCAE toxicity between pretreatment (PT) and one-year follow-up (1Y).

| **CTCAE** |  | **Adjusted* p-value** |
| --- | --- | --- |
| **Systemic** |  |  |
| Fatigue |  | 0.06 |
| Pain |  | 0.68 |
| **Gastrointestinal** |  |  |
| Anorexia |  | 0.13 |
| Bloating |  | 0.62 |
| Constipation |  | 0.24 |
| Diarrhoea |  | < 0.01 |
| Flatulence |  | 0.47 |
| Nausea |  | 1.00 |
| Vomiting |  | 0.44 |
| Rectal/stool haemorrhage |  | <0.001 |
| **Urogenital** |  |  |
| Haematuria |  | 0.41 |
| Urinary frequency |  | 0.01 |
| Urinary incontinence |  | <0.01 |
| Urinary tract pain |  | 0.47 |
| Urinary retention |  | <0.001 |
| Urinary urgency |  | <0.01 |
| **Other** |  |  |
| Radiation dermatitis |  | 0.10 |
| PPI |  | 0.15 |

*The Wilcoxon signed-rank test was applied to each toxicity domain. Given the multiple comparisons performed across different toxicity outcomes, the Benjamini-Hochberg method was used to adjust for multiple testing.
